# Deep medullary vein damage correlates with small vessel disease in small vessel occlusion acute ischemic stroke

**DOI:** 10.1007/s00330-024-10628-4

**Published:** 2024-02-10

**Authors:** Xueyang Wang, Jinhao Lyu, Qi Duan, Chenxi Li, Jiayu Huang, Zhihua Meng, Xiaoyan Wu, Wen Chen, Guohua Wang, Qingliang Niu, Xin Li, Yitong Bian, Dan Han, Weiting Guo, Shuai Yang, Xiangbing Bian, Yina Lan, Liuxian Wang, Tingyang Zhang, Caohui Duan, Xin Lou, on behalf of the MR-STARS Investigators

**Affiliations:** 1 Department of Radiology, Chinese PLA General Hospital, Beijing, China; 2 Department of Radiology, Yancheng Traditional Chinese Medicine Hospital Affiliated to Nanjing University of Chinese Medicine/ Yancheng Traditional Chinese Medicine Hospital, Jiangsu, China; 3 Department of Radiology, Yuebei People’s Hospital, Guangdong, China; 4 Department of Radiology, Anshan Changda Hospital, Liaoning, China; 5 Department of Radiology, Shiyan Taihe Hospital, Hubei, China; 6 Department of Radiology, Qingdao Municipal Hospital Affiliated to Qingdao University, Shandong, China; 7 Department of Radiology, WeiFang Traditional Chinese Hospital, Shandong, China; 8 Department of Radiology, Jilin University Second Hospital, Shandong, China; 9 Department of Radiology, The First Affiliated Hospital of Xi’an Jiaotong University, Shaanxi, China; 10 Department of Radiology, Kunming Medical University First Affiliated Hospital, Yunnan, China; 11 Department of Radiology, Shanxi Provincial People’s Hospital, Shanxi, China; 12 Department of Radiology, Xiangya Hospital Central South University, Hunan, China

**Keywords:** Deep medullary vein, Cerebral small vessel disease, Perivascular space, Stroke

## Abstract

**Objectives:**

We aim to investigate whether cerebral small vessel disease (cSVD) imaging markers correlate with deep medullary vein (DMV) damage in small vessel occlusion acute ischemic stroke (SVO-AIS) patients.

**Methods:**

The DMV was divided into six segments according to the regional anatomy. The total DMV score (0–18) was calculated based on segmental continuity and visibility. The damage of DMV was grouped according to the quartiles of the total DMV score. Neuroimaging biomarkers of cSVD including white matter hyperintensity (WMH), cerebral microbleed (CMB), perivascular space (PVS), and lacune were identified. The cSVD score were further analyzed.

**Results:**

We included 229 SVO-AIS patients, the mean age was 63.7 ± 23.1 years, the median NIHSS score was 3 (IQR, 2–6). In the severe DMV burden group (the 4th quartile), the NIHSS score grade (6 (3–9)) was significantly higher than other groups (*p* < 0.01). The grade scores for basal ganglia PVS (BG-PVS) were positively correlated with the degree of DMV (*R* = 0.67, *p* < 0.01), rather than centrum semivole PVS (CS-PVS) (*R* = 0.17, *p* = 0.1). In multivariate analysis, high CMB burden (adjusted odds ratio [aOR], 25.38; 95% confidence interval [CI], 1.87–345.23) was associated with severe DMV scores. In addition, BG-PVS was related to severe DMV burden in a dose-dependent manner: when BG-PVS score was 3 and 4, the aORs of severe DMV burden were 18.5 and 12.19, respectively.

**Conclusion:**

The DMV impairment was associated with the severity of cSVD, which suggests that DMV burden may be used for risk stratification in SVO-AIS patients.

**Clinical relevance statement:**

The DMV damage score, based on the association between small vessel disease and the deep medullary veins impairment, is a potential new imaging biomarker for the prognosis of small vessel occlusion acute ischemic stroke, with clinical management implications.

**Key Points:**

*• The damage to the deep medullary vein may be one mechanism of cerebral small vessel disease.*

*• Severe burden of the basal ganglia perivascular space and cerebral microbleed is closely associated with significant impairment to the deep medullary vein.*

*• The deep medullary vein damage score may reflect a risk of added vascular damage in small vessel occlusion acute ischemic stroke patients.*

**Supplementary Information:**

The online version contains supplementary material available at 10.1007/s00330-024-10628-4.

## Introduction

Stroke is the leading cause of disability in the world and a frequent cause of death [1]. Among acute ischemic stroke (AIS), there are different subtypes according to TOAST, including large artery atherosclerosis (LAA), cardioembolism, and small vessel occlusion (SVO) [2]. Currently, there is a significant focus on conducting in-depth research on LAA-AIS pathologies, whereas a notable scarcity of research dedicated to investigating the prognosis of patients with SVO-AIS remains [3]. Traditionally, SVO subtype stroke is thought to result from disease of a small perforating artery and manifests with several syndromes, depending on lesion location in clinical, while on imaging, the appearance of sequelae of intracranial arterioles and venules injury is usually described by lacunes, white matter hyperintensity (WMH), perivascular space (PVS), and cerebral microbleed (CMB). With improved cognition, these imaging findings can provide data on additional pathophysiological mechanisms involved in SVO subtype stroke, thus providing scientific basis for developing specific treatment strategies.

On conventional MR image, the large intracranial arteries were usually evaluated on three-dimensional time-of-flight magnetic resonance angiography (3D TOF-MRA), whereas with the limitation of definition, arterioles were poorly displayed [4]. For the instruments displaying intracranial veins, through exploiting the susceptibility effect from deoxyhemoglobin in veins, susceptibility-weighted imaging (SWI) sequence has been recognized as a sensitive method for visualizing venules in vivo [5, 6]. The cerebral venous system can be divided into a superficial and a deep system [7]. The deep medullary vein (DMV) plays a crucial role in draining the venous blood from the deep brain tissue, facilitating the removal of metabolic waste and maintaining optimal cerebral blood flow [8, 9]. Simultaneously, the nuclei nestled deep within the brain tissue are central to regulating critical brain functions, such as cognition, movement, and sensation, ensuring proper neurological processing and integration [10]. Thus, the DMV may represent essential components of the complex neural network. Moreover, the DMV can be relatively easily assessed owing to their consistent anatomical trajectory and perpendicular to the lateral ventricles [11]. Several studies have demonstrated that the disruption in continuity or reduction in quantity of DMV on SWI sequence may represent the development of venous collagenosis (VC), which is thought to play a role in the pathogenesis of cerebral small vessel disease [12, 13].

To date, due to the lack of studies, DMV damage has been integrated into cerebrovascular disease and little is known about the potential risk factors related to changes in DMV. Moreover, there is a need for in-depth exploration into the potential association between pathological changes in DMV, such as discontinuity, or heterogeneous signal seen on SWI, and the development of WMH, PVS, CMB, and other imaging markers of cSVD in the population with SVO-AIS. Additionally, the possibility of developing DMV characteristics as an indicator for evaluating SVO-AIS patients warrants further investigation.

The purpose of this study is to explore the continuity and signal features of DMV on SWI phase images in individuals with SVO-AIS, as well as to examine the potential differences in DMV characteristics between patients with varying DMV grade scores. Additionally, this study aims to investigate the potential correlation between severe DMV burden and the presence of imaging markers of SVD, such as PVS, CMB, and lacunes.

## Materials and methods

### Patients

We retrospectively evaluated patients from a multicenter registry study conducted in 11 centers (MR ClinicalTrials.gov Identifier: NCT02580097) from January 2019 to December 2020. This is a population-based study of all stroke events occurring within 24 h, which consisted of 961 individuals. All enrolled patients were classified according to the TOAST classification standard, and SVO-AIS patients were included in the analysis. The screening criteria for SVO-AIS were as follows: (1) the patient’s intracranial major arteries were normal; (2) the diffusion-weighted imaging (DWI) sequence showed high punctate signals, with stroke diameter under 15 mm; (3) other causes of infarction were excluded. Magnetic resonance images of all patients were the initial examination images taken within 12 h of their admission after the onset of the symptoms, and received standard medical treatment.

For the current study, the patients also met the additional inclusion criteria: (1) MR protocol including T1-weighted imaging (T_1_WI), T2-weighted imaging (T_2_WI), T_2_FLAIR, DWI, SWI, and MRA; (2) MR imaging met the Standards for Reporting Vascular changes on Neuroimaging (STRIVE) for SVD; the exclusion criteria were (1) any MRI contraindications; (2) incomplete baseline data; (3) presence of other brain abnormalities such as tumors, infection, trauma, acute hemorrhagic infarction, or chronic infarction; and (4) patients with hereditary cSVD. This research was approved by the ethics committee authorities in all participating groups, and written informed consent was obtained from patients.

### Clinical information

Demographic and clinical data were collected through a multicenter and local hospital dataset. The following stroke risk factors were identified: age, sex, hypertension, hyperglycemia, hyperlipidemia, coronary heart disease, atrial fibrillation, smoking, and alcohol consumption. During the case collection process, patients with missing clinical data were excluded from the study.

### MR protocol

MRI was performed with a 3.0-T MRI scanner (Discovery MR750; General Electric) using a 32-channel phased-array head coil. The imaging protocol with fat saturation included three-dimensional time-of-flight MRA (3D TOF-MRA), DWI, T1-weighted fast spin echo (T_1_WFSE), T2-weighted FSE (T_2_WFSE), T2 fluid attenuated inversion recovery (T_2_ FLAIR), and SWI. The relevant important parameters were SWI: TR/TE = minimum/25 ms, FOV = 240 × 240 mm^2^, matrix = 320 × 224, slice thickness = 2 mm, slices = 282, flip angle = 15°, bandwidth = 62.5 Hz/pixel.

### Measurement of DMVs

**Fig. 1:**
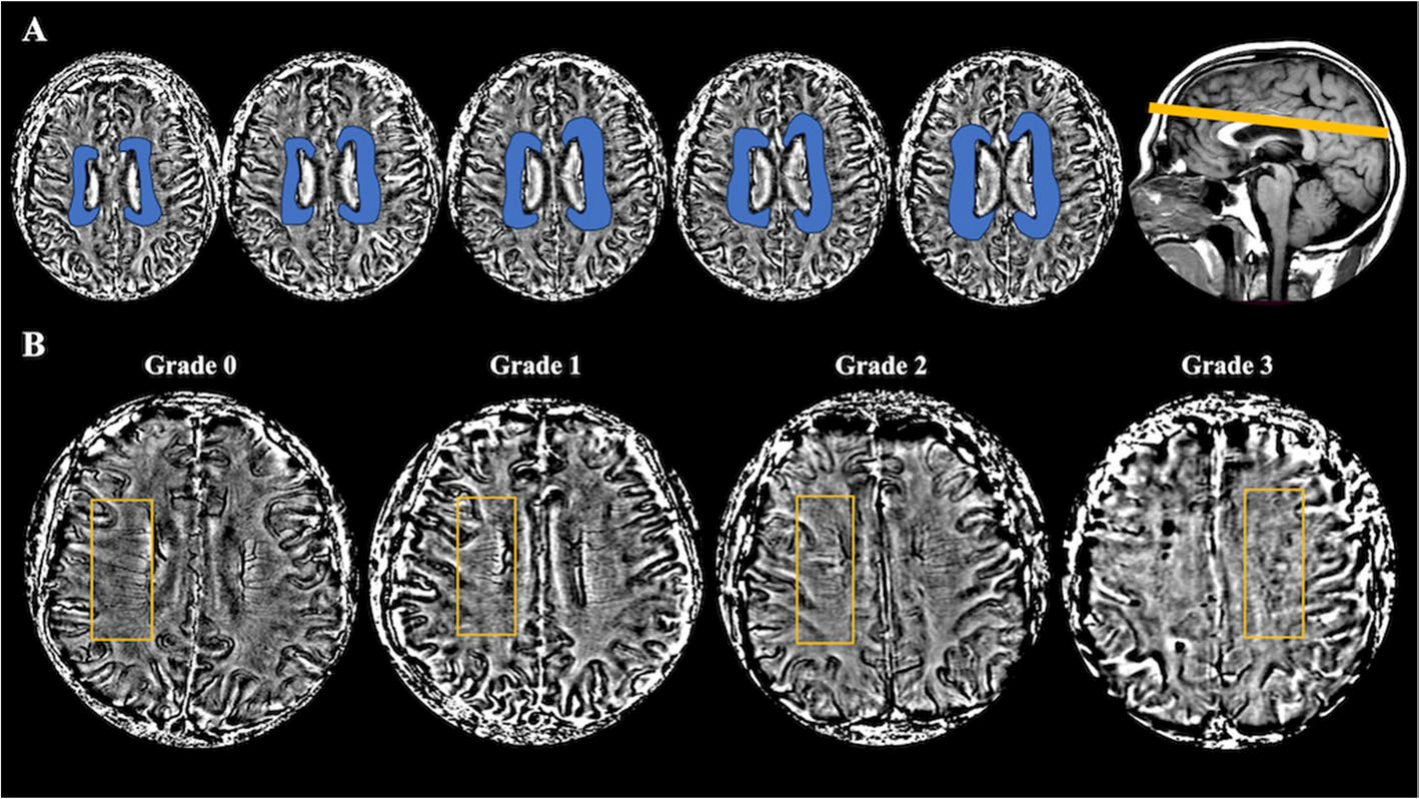
**A** The colored areas were deep medullary veins (DMVs) of three different brain regions including frontal region, parietal region, and occipital region according to medullary venous anatomy. **B** DMVs visual scores. An example of four-point DMV score in parietal region: grade 0—each vein was continuous and had homogeneous signal; grade 1—each vein was continuous, but one or more than one vein had inhomogeneous signal; grade 2—one or more than one vein was not continuous, presented with spot-like hypointensity; grade 3—no observed vein was found continuous

DMV scores were assessed according to the SWI sequences. We evaluated five consecutive SWI-phase periventricular slices (2 × 5 mm thick) from superior basal ganglia to the level of the ventricles, considering that these slices cover most of the DMV (Fig. 1A).

The deep medullary veins were divided into 6 regions, including frontal, parietal, and occipital (bilateral, respectively), by a method of venous assessment that has been widely used in previous studies [14, 15]. Each region was scored separately with a DMV score 0–3 for the continuity and integrity of DMV. The DMV score 0 indicates that each vein is continuous, uninterrupted, and significantly visible. A score of 1 means that all the DMVs are continuous and prominently visible, but at least one vein is present with an inhomogeneous signal. A score of 2 indicates that at least one vein is discontinuous with diminished signal, presenting with punctuated hypointensity. A score of 3 is assigned when the DMVs are poorly visualized, and no continuous venous is visible in the segments (Fig. 1B). Therefore, the total DMV score was calculated as 0–18 and introduced into the subsequent analysis.

All images were reviewed individually by two radiologists (X.Y.W. and Q.D. with 12 and 5 years of experience in neuroradiology, respectively) who were completely blinded to the subjects’ clinical data and disease status. Disagreements were resolved by a senior radiologist (J.H.L. with 14 years of experience in neuroradiology).

### Evaluation of SVD imaging markers

The SVD burden was assessed by two radiologists (X.Y.W. and Q.D.) who were blinded to the clinical history, patient identity, and prognosis of the patients. Any disparity was resolved by a senior radiologist (J.H.L.). The identification of SVD imaging markers, such as chronic lacune, CMB, WMH, and PVS, was based on the STandards for ReportIng Vascular changes on nEuroimaging (STRIVE) [16]. WMH was differentiated into periventricular WMH (P-WMH) and deep WMH (D-WMH), and the severity of WMH was assessed separately using Fazekas scoring system [17]. The PVS was graded in the slice which contained the maximum PVS in the basal ganglia or the centrum semiovale, and if there was any asymmetry, the worse side was selected [18].

To calculate the total SVD score, we evaluated chronic lacune, CMB, WMH, and PVS based on the ordinal scale from 0 to 4, which has been widely used by several studies [18, 19]. The modified total SVD score (0–6) was also applied in the present study to assess the following: presence of lacunes, 1 point; 1–4 CMBs, 1 point; ≥ 5 CMBs, 2 points; > 20 basal ganglia PVS (BG-PVS), 1 point; moderate WMH (total Fazekas = 3–4), 1 point; and severe WMH (total Fazekas = 5–6), 2 points [20].

### Statistical analysis

The primary outcomes were the degree of scoring of DMV, and the main dependent variables were the imaging markers of SVD (WMH, PVS, chronic lacunes, and CMB). Quantitative data were presented as means ± standard deviations, according to the normality of the distribution of continuous variables, and categorical data were described as percentages. Baseline characteristics of the overall cohort were described and compared across patients with different levels of DMVs using analysis of variance or continuous variables and chi-square test for categorical variables. The prevalence of SVD subtypes including chronic lacunes and CMB, as well as the PVS semi-quantitative score from 1 to 4 and WMH Fazekas scale score from 0 to 3, were also described and compared between patients with different degrees of DMVs using the Mann–Whitney *U* test or chi-square test. Bonferroni tests were used for correction. Multivariable logistic regression was performed to examine the relationship between SVD and DMV by calculating the odds of heavy DMV burden before and after adjusting for age, sex, initial National Institute of Health stroke (NIHSS) score, alcohol consumption, smoking, hypertension, hyperglycemia, hyperlipidemia, atrial fibrillation (AF), and coronary heart disease (CHD). The dose-dependent trends were tested in the D-WMH or P-WMH (Fazekas scale 0–3) and BG-PVS or centrum semiovale PVS (CS-PVS) (semiquantitative scale 1–4). The simple Cohen kappa statistic was used for the assessment of different SVD imaging markers. All statistical analyses were performed using SPSS version 26.0. (IBM Corporation).

## Results

### Patient characteristics

**Fig. 2:**
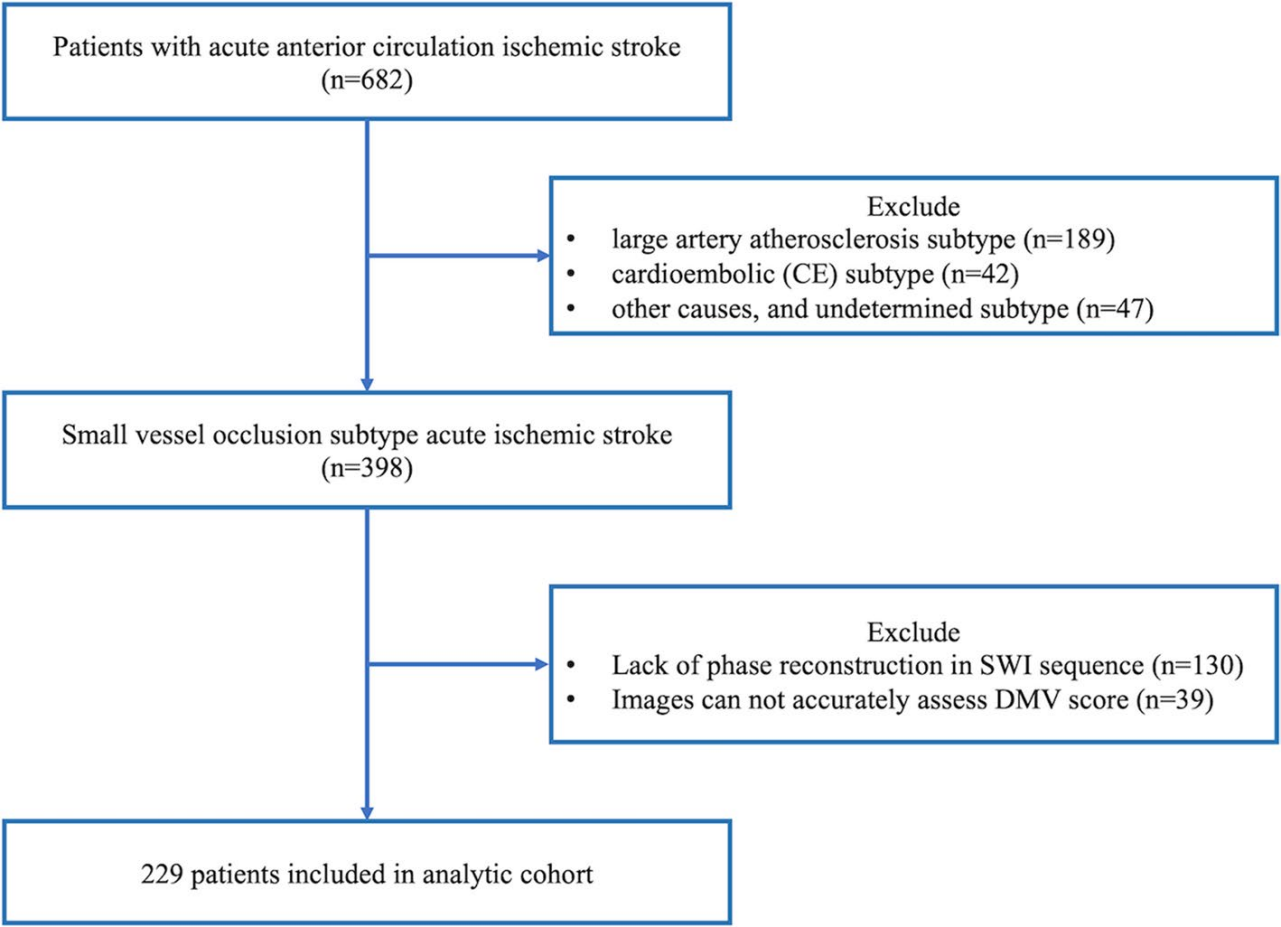
SWI, susceptibility weight imaging; DMV, deep medullary vein

A total of 398 patients with SVO-AIS patients, screened from 682 cases of acute anterior ischemic stroke, were enrolled in the current study (Fig. 2). Among these, 130 patients were excluded due to lack of SWI phase image reconstruction, and 39 patients were ruled out due to poor image definition and could not be accurately evaluated. Finally, a total of 229 patients were included in the final analysis, with an average age of 63.7 ± 23.1 years, and 29.3% were women. All patients were divided into four groups according to quartiles of DMV scores: 1st quartile (DMV score 0–2, *n* = 64), 2nd quartile (DMV score 3–6, *n* = 72), 3rd quartile (DMV score 7–10, *n* = 42), 4th quartile (DMV score 11–18, *n* = 51). Age and initial admission NIHSS score significantly differed among different groups based on the degree of DMV score. Patients with higher DMV scores (3rd and 4th quartile) had older mean age (72.6 ± 45.9 years old and 71.9 ± 9.1 years old) than 1st and 2nd quartile (*p* < 0.01). In the most severe DMV burden group (the 4th quartile), the initial admission NIHSS score grade (6 (3–9)) was significantly higher than that of other groups (*p* < 0.01). In contrast, there were no remarkable differences in vascular risk factors including hypertension, hyperlipidemia, and hyperglycemia among patients in different DMV score group. (Table 1).

**Table 1:** Patient characteristics and prevalence of SVD characteristics stratified by DMV score quartile

	The score of DMV					
	Overall	1st quartile (0–2)	2nd quartile (3–6)	3rd quartile (7–10)	4th quartile (11–18)	*p* value
	*n* = 229	*n* = 64	*n* = 72	*n* = 42	*n* = 51	
Demographics						
Female sex, *n* (%)	67 (29.3)	23.4	23.6	35.7	39.2	0.14
Age, year; mean ± SD	63.7 ± 23.1	54.3 ± 12.8	60.9 ± 10.4	72.6 ± 45.9	71.9 ± 9.1	< 0.01
Initial NIHSS at 24 h, median (IQR)	3 (2–6)	3(2–5)	2.5(2–4)	3(2–4.5)	6(3–9)	< 0.01
Medical history, *n* (%)						
Hypertension	147 (64.2)	36 (56.3)	46 (63.9)	29 (69)	36 (70.6)	0.38
Hyperglycemia	100 (43.7)	26 (40.6)	27 (37.5)	18 (42.9)	29 (56.9)	0.17
Hyperlipemia	88 (38.4)	29 (45.3)	25 (34.7)	16 (38.1)	18 (35.3)	0.59
CHD	34 (14.8)	8 (12.5)	11 (15.3)	6 (14.3)	9 (17.6)	0.89
AF	16 (7)	3 (4.7)	2 (2.8)	4 (9.5)	7 (13.7)	0.09
Ever smoker	91 (39.7)	25 (39.1)	34 (47.2)	13 (31)	19 (37.3)	0.36
Alcohol	61 (26.6)	19 (29.7)	20 (27.8)	10 (23.8)	12 (23.5)	0.86

*SVD* small vessel disease, *DMV* deep medullary vein, *IQR* inter-quartile range, *CHD* coronary heart disease, *AF* atrial fibrillation

### Association of DMV scores with chronic SVD burden

SVD imaging markers for the different degree of DMV subgroups are presented in Table 2. In the univariate analysis, the 4th quartile subgroup had the highest proportion of patients with chronic lacunes (*p* < 0.01). The BG-PVS score had the highest Spearman’s correlation coefficient (*R* = 0.67), indicating a strong connection with the continuity and signal of the DMV. However, the grade score of CS-PVS did not correlate with the DMV score (*R* = 0.17, *p* = 0.1). In addition, the scores of CMB, P-WMH, and D-WMH were also strongly correlated with the scores of DMV (*R*-values were 0.41, 0.58, and 0.61, respectively); however, the extent of the association was weaker than that of BG-PVS.

**Table 2:** Prevalence of SVD characteristics and severity of SVD stratified by degree of DMV scores

	The score of DMV						
	Overall	1st quartile (0–2)	2nd quartile (3–6)	3rd quartile (7–10)	4th quartile (11–18)	*R* value	*p* value
Presence of SVD, *n* (%)	*n* = 229	*n* = 64	*n* = 72	*n* = 42	*n* = 51		
Lacune	87 (38)	6 (9.4)	17 (23.6)	22 (52.4)	42 (82.4)	0.56	< 0.01
CMB						0.41	< 0.01
0 (*n* = 0)	157 (68.6)	54 (84.4)	60 (83.3)	22 (52.4)	21 (41.2)		
1 (*n* = 1–4)	47 (20.5)	10 (15.6)	11 (15.3)	13 (31)	13 (25.5)		
2 (*n* ≥ 5)	25 (10.9)	0 (0)	1 (1.4)	7 (16.7)	17 (33.3)		
P-WMH						0.58	< 0.01
Fazekas scale 0	22 (9.6)	15 (23.4)	6 (8.3)	1 (2.4)	0 (0)		
Fazekas scale 1	107 (46.7)	39 (60.9)	43 (59.7)	18 (42.9)	7 (13.7)		
Fazekas scale 2	56 (24.5)	8 (12.5)	19 (26.4)	15 (35.7)	14 (27.5)		
Fazekas scale 3	44 (19.2)	2 (3.1)	4 (5.6)	8 (19)	30 (58.8)		
D-WMH						0.61	< 0.01
Fazekas scale 0	18 (7.9)	12 (18.8)	6 (8.3)	0 (0)	0 (0)		
Fazekas scale 1	131 (57.2)	49 (76.6)	48 (66.7)	25 (59.5)	9 (17.6)		
Fazekas scale 2	41 (17.9)	2 (3.1)	14 (19.4)	10 (23.8)	15 (29.4)		
Fazekas scale 3	39 (17)	1 (1.6)	4 (5.6)	7 (16.7)	27 (52.9)		
CS-PVS						0.17	0.10
Semiquantitative score 1	68 (29.7)	26 (40.6)	23 (31.9)	11 (26.2)	8 (15.7)		
Semiquantitative score 2	75 (32.8)	16 (25)	23 (31.9)	16 (38.1)	20 (39.2)		
Semiquantitative score 3	59 (25.8)	16 (25)	21 (29.2)	10 (23.8)	12 (23.5)		
Semiquantitative score 4	27 (11.8)	6 (9.4)	5 (6.9)	5 (11.9)	11 (21.6)		
BG-PVS						0.67	< 0.01
Semiquantitative score 1	97 (42.4)	49 (76.6)	38 (52.8)	10 (23.8)	0 (0)		
Semiquantitative score 2	55 (24)	12 (18.8)	23 (31.9)	11 (26.2)	9 (17.6)		
Semiquantitative score 3	24 (10.5)	2 (3.1)	3 (4.2)	15 (35.7)	4 (7.8)		
Semiquantitative score 4	53 (23.1)	1 (1.6)	8 (11.1)	6 (14.3)	38 (74.5)		

*SVD* small vessel disease, *DMV* deep medullary vein, *CMB* cerebral microbleed, *P-WMH* periventricular white matter hyperintensity, *D-WMH* deep white matter hyperintensity, *CS-PVS* centrum semiovale perivascular space, *BG-PVS* basal ganglia perivascular space

In the multivariate analysis, covariates such as age, sex, initial NIHSS score, alcohol consumption, smoking, hypertension, hyperglycemia, hyperlipidemia, atrial fibrillation, and chronic heart disease were adjusted. There was a dose-dependent relationship between BG-PVS and heavy DMV burden; aORs of heavy DMV burden were 18.5 and 12.19, respectively, across semi-quantitative scores PVS of 3–4 (Table 3, Supplement Fig. 1, Supplement Fig. 2). However, the PVS in the centrum semiovale were not correlated with each other. Heavy burden of DMV continued to be more likely in patients with severe CMB burden (the number of CMB is more than or equal to 5), the aOR was 25.38, and 95% confidence interval was 1.87–345.23.

**Table 3:** Prevalence and odds of heavy DMV burden by different SVD subtypes

	Prevalence of heavy DMV burden, *n* (%)	Crude		Adjust	
		OR	*p*	OR	*p*
P-WMH					
Fazekas scale 0	1 (4.5)	REF		REF	
Fazekas scale 1	25 (23.4)	0.97 (0.11–8.38)	0.98	0.66 (0.06–6.88)	0.73
Fazekas scale 2	29 (51.8)	0.92 (0.80–10.09)	0.95	0.54 (0.04–7.44)	0.65
Fazekas scale 3	38 (86.3)	0.53 (0.02–11.97)	0.69	0.19 (0.01–5.40)	0.33
* p* trend			0.96		0.76
D-WMH					
Fazekas scale 0	0 (0)	N/A		N/A	
Fazekas scale 1	34 (26.0)	REF		REF	
Fazekas scale 2	25 (61)	0.98 (0.29–3.30)	0.97	0.72 (0.19–2.71)	0.62
Fazekas scale 3	34 (87.2)	3.51 (0.40–30.80	0.26	5.76 (0.52–63.26)	0.15
* p* trend			0.67		0.37
CS-PVS					
Semiquantitative score 1	19 (27.9)	REF		REF	
Semiquantitative score 2	36 (48)	1.26 (0.49–3.28)	0.64	1.41 (0.49–4.00)	0.524
Semiquantitative score 3	22 (37.3)	0.58 (0.20–1.70)	0.32	0.73 (0.23–2.37)	0.6
Semiquantitative score 4	16 (59.3)	0.70 (0.17–2.91)	0.63	0.78 (0.17–3.54)	0.75
* p* trend			0.46		0.67
BG-PVS					
Semiquantitative score 1	10 (10.3)	REF		REF	
Semiquantitative score 2	20 (36.4)	3.26 (1.21–8.79)	0.02	2.72 (0.90–8.24)	0.08
Semiquantitative score 3	19 (79.2)	15.00 (4.02–63.60)	< 0.01	18.50 (3.94–86.31)	< 0.01
Semiquantitative score 4	44 (83.0)	12.33 (3.31–45.93)	< 0.01	12.19 (2.66–55.92)	< 0.01
* p* trend			< 0.01		< 0.01
CMB					0.02
0 (*n* = 0)	43 (27.4)	REF		REF	
1 (*n* = 1–4)	26 (55.3)	1.35 (0.55–3.34)	0.51	1.18 (0.44–3.16)	0.74
2 (*n* ≥ 5)	24 (96)	14.14 (1.33–150.23)	0.03	25.38 (1.87–345.23)	0.02
* p* trend			0.09		0.05
Lacune	64 (73.6)	2.03 (0.77–5.33)	0.15	1.65 (0.55–4.98)	0.38

*DMV* deep medullary vein, *SVD* small vessel disease, *P-WMH* periventricular white matter hyperintensity, *D-WMH* deep white matter hyperintensity, *CS-PVS* centrum semiovale perivascular space, *BG-PVS* basal ganglia perivascular space, *CMB* cerebral microbleed

### Association of DMV scores with total SVD burden

**Fig. 3:**
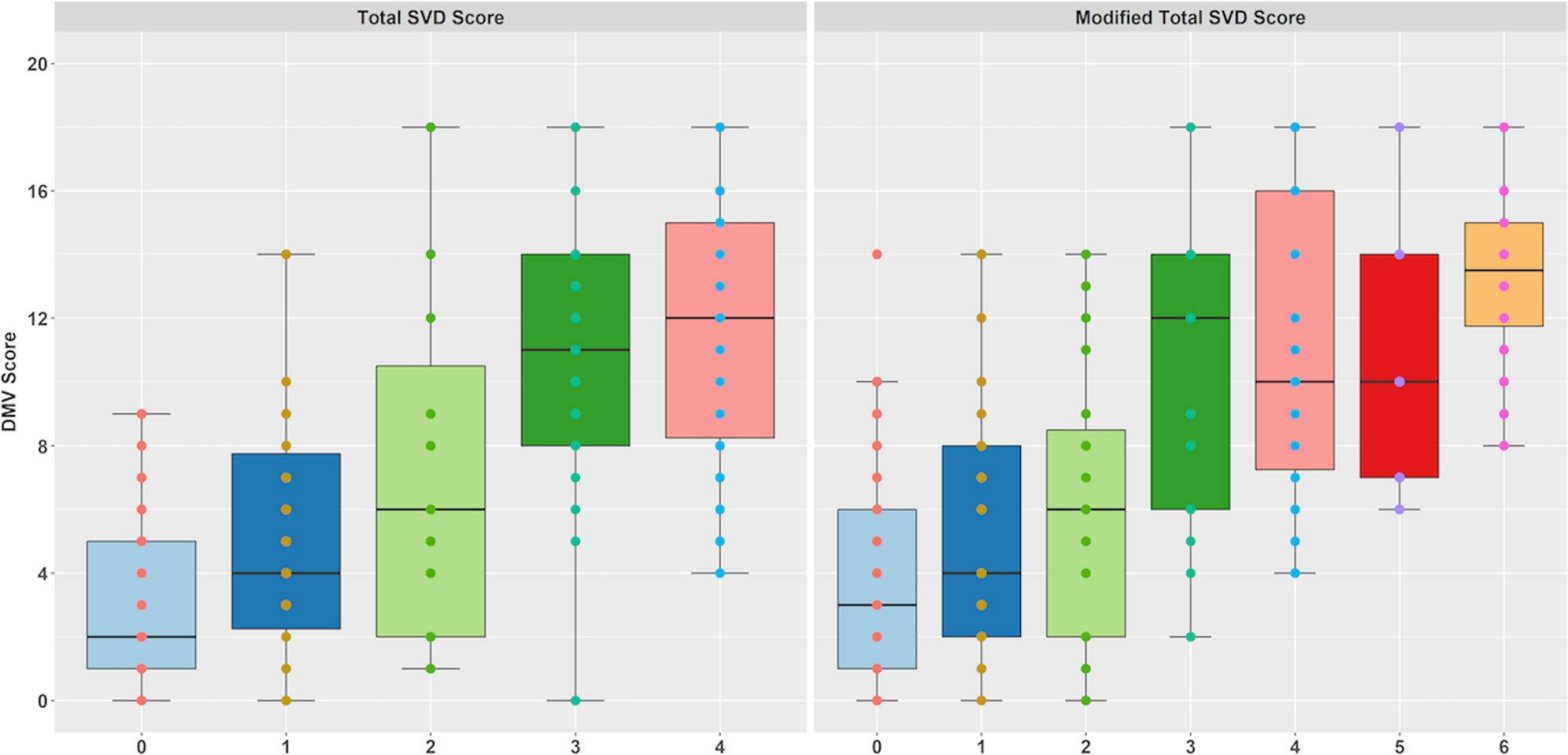
The correlation between different types of total SVD score and DMV score. SVD, small vessel disease; DMV, deep medullary vein

Among all the 229 patients, the total SVD burden was also evaluated. Total SVD score (4-point scale) and modified SVD score (6-point scale) were introduced, respectively. In Fig. 3, box plot shows an increasing trend in DMV scores as the patients’ SVD burden progressed.

### Interobserver assessment

There was excellent interobserver agreement in evaluating the DMV score, P-WMH, D-WMH, BG-PVS, CS-PVS, lacunes, and CMB, with Kappa values of 0.785, 0.852, 0.879, 0.834, 0.856, 0.821, and 0.89, respectively.

## Discussion

In this study of patients with SVO-AIS patients, there was a correlation between DMV and chronic SVD burden, especially in CMB and BG-PVS; however, the severity of CS-PVS and WMH (either periventricular WMH or deep WMH) were not the risk factors for DMV impairment. This suggested that venous disruption may be involved in the pathogenesis of BG-PVS and CMB. In LAA-AIS patients, the impaired venous drainage and disruption of DMV have been found to result in decreased oxygen and nutrient supply to affected brain tissue, contributing to the progression of ischemic injury [21, 22]. However, in patients with SVO, the burden of SVD can serve as one of the predictors for prognosis in SVO-AIS patients [23, 24]. Therefore, the study of the correlation between SVD burden and DMV visualization status is of wide concern in the field of cerebral vascular disease.

The SVO-AIS is clinically insidious and atypical [25]. It appears on MR as DWI sequence of punctate high signal and is not accompanied by stenosis or occlusion of intracranial large arteries; however, it is usually present with WMH, PVS, CMB, and other SVD imaging markers [25]. Although the total SVD burden alone summarizes the overall changes to brain tissue and indirectly reflects the pathological development at the level of small blood vessels, these markers lack visual representation of the intracranial microcirculation. With the application of SWI sequence, it is feasible to demonstrate veins without any contrast agent. The DMV is the main vein for draining the tissue fluid from deep white matter and runs horizontally, which is easy to observe in SWI sequence [15]. Intuitive assessment of DMV and SVD burden may enhance understanding of the microcirculation status. Therefore, DMV can be considered an important image marker for clinical prognosis evaluation in SVO-AIS patients.

Previously, the mechanisms of different SVD imaging markers and their impact on clinical prognosis have been investigated in depth [26, 27]. Among them, PVS has been proven to be the essential structure of the glymphatic pathway in the brain. This route facilitates the flow of cerebrospinal fluid (CSF) into the brain tissue along the PVS surrounding the arterioles and into the interstitium to exchange with interstitial fluid (ISF). The pathway then drains the exchanged ISF into PVS around venules, which ultimately removes the “garbage” into meningeal and cervical lymphatic system [28]. Since the presence of pathophysiologic mechanisms, the heavy burden of PVS indicates a blockage of the glymphatic fluid stasis, which leads to increased static pulse pressure, perivenular edema, and venous wall thickening, stenosis, and occlusion, resulting in reduced visibility and continuous disruption of venules on SWI sequence [29, 30]. Interestingly, in the present study, for SVO-AIS patients, the damage to the venous was related to the presence of BG-PVS, rather than CS-PVS. A conceivable explanation lies in the fact that the basal ganglia region is drained by DMVs only, whereas the centrum semiovale area can be drained by cortical and superficial medullary veins additionally [15]. Furthermore, the penetrating arteries in the basal ganglia region are nearly vertically bifurcated, making them susceptible to vascular risk factors. This condition may also lead to damage to arterioles, reduced clearance of glymphatic, and even injuries to venules. We assume that both interpret the correlation between the PVS in basal ganglia region and DMV injury.

Moreover, with respect to the correlation between SVD and DMV, CMB also attracted some other researchers’ attention. The pathogenesis of CMB is currently unclear and there are many hypotheses, including hypoperfusion damage, impaired blood-brain barrier (BBB), inflammatory response, endothelial damage, β-amyloid deposition, and genetic polymorphisms. And nowadays, the hypothesis of BBB impairment as the mechanism of CMB occurrence has been validated in both autopsy and animal models [30, 31]. Besides, it has been verified before that high DMV score may indicate the disruption of BBB [32]. These theoretical explanations are pertinent to the results of our study, in which the number of CMB was graded using a method from the modified total SVD score, and the study suggested a strong correlation between heavy CMB burden (the number of CMB above or equal to 5) and DMV injury. This observation further implicates that SWI sequences showing above or equal to 5 CMBs were more reflective of microvascular damage and increased BBB permeability, also implying a poor prognosis.

In integrating the above pathological and hemodynamic changes of SVD, we believe the DMV plays a key role in the formation and progression of SVD; however, the present study revealed no connection between WMH and severe DMV burden. Some discrepancies exist in previous studies regarding the association of WMH and DMV. In 2014, Yan et al found that the patients with WMH had higher DMV volumes than healthy controls [14]. Meanwhile, a 2020 study discovered that DMV and WMH were strongly correlated. However, this inverse correlation between the number of DMV and volume of WMH vanished after adjustment for age [33]. Thus, we consider the changes in DMV associated with WMH to be a gradual progression. At an earlier stage, DMV was present prominently due to increased deoxyhemoglobin, elevated venous pressure, and compensatory dilatation. With progression of WMH, DMV changed from compensatory dilatation to venous collagenous and venous lumen altered from stenosis to even complete occlusion. Therefore, the status of the venules can be used to indicate the different stages of WMH, thus facilitating stratification of patients.

In terms of chronic lacunes, similar to WMH, no relationship was found with DMV. Although lacunes differed among patients with different DMV score, it is not a risk factor for heavy DMV burden in our research. This finding may have some relevance to the enrolled SVO-AIS population, which had an older age and more vascular risk factors. Moreover, in the present study, we not only explored its correlation with each imaging biomarker of SVD according to STRIVE, but also two total SVD burden scoring methods were further employed to investigate the impact of SVD burden on DMV visibility.

There were some limitations in our study. This study lacked an assessment of brain atrophy, another important imaging marker of SVD, which is closely associated with age. We have attempted to adopt a template based on a normal elderly population for visual score analysis the morphology of the ventricular system and the cortical sulci but did not obtain good interobserver correlation coefficients. Therefore, a 3D T1-weighted sequence using computer segmentation processing is needed in the future to quantitatively observe alterations in brain volume. In addition, in terms of brain imaging data analysis, the assessment of WMH and PVS should have both subjective and objective components. Furthermore, in the evaluation of DMV scores, although SWI sequence in 3 T may get a relatively high-definition image, 7 T MRI of quantitative DMV research are imperative in the future, reducing the probability of misestimate.

## Conclusion

This study found that DMV disruption was associated with chronic SVD burden, especially BG-PVS and severe CMB burden independent of age, hypertension, and other vascular risk factors, revealing that venous dysfunction participates in the pathogenesis of SVD. In clinical practice, the presence of DMV in SWI sequence allows visualization of the state of intracranial microcirculation; as a result, close observation of DMV may be helpful in the assessment of the progression and prognosis of SVO-AIS patients. However, further longitudinal studies are warranted in the future.

### Supplementary Information

Below is the link to the electronic supplementary material.

Supplementary file1 (PDF 343 KB)

## Abbreviations

AIS: Acute ischemic stroke
BBB: Blood-brain barrier
CMB: Cerebral microbleed
cSVD: Cerebral small vessel disease
DMV: Deep medullary vein
DWI: Diffusion-weighted imaging
MRA: Magnetic resonance angiogram
PVS: Perivascular space
SVO-AIS: Small vessel occlusion acute ischemic stroke
SWI: Susceptibility-weighted imaging
WMH: White matter hyperintensity

## Declarations

### Guarantor

The guarantor of this study is Xin Lou.

### Statistics and biometry

No complex statistical methods were necessary for this paper.

### Methodology

• retrospective

• cross sectional study

• performed at multicenter
