# Supplementary material for: Deep medullary vein damage correlates with small vessel disease in small vessel occlusion acute ischemic stroke
Source: Eur Radiol. 2024 Feb 10;34(9):6026–35. doi: 10.1007/s00330-024-10628-4 (PMC11364723; doi:10.1007/s00330-024-10628-4)
Supplement: Supplementary file 1 — Supplementary file1 (PDF 343 KB) [file 330_2024_10628_MOESM1_ESM.pdf]

Supplement Figure 1

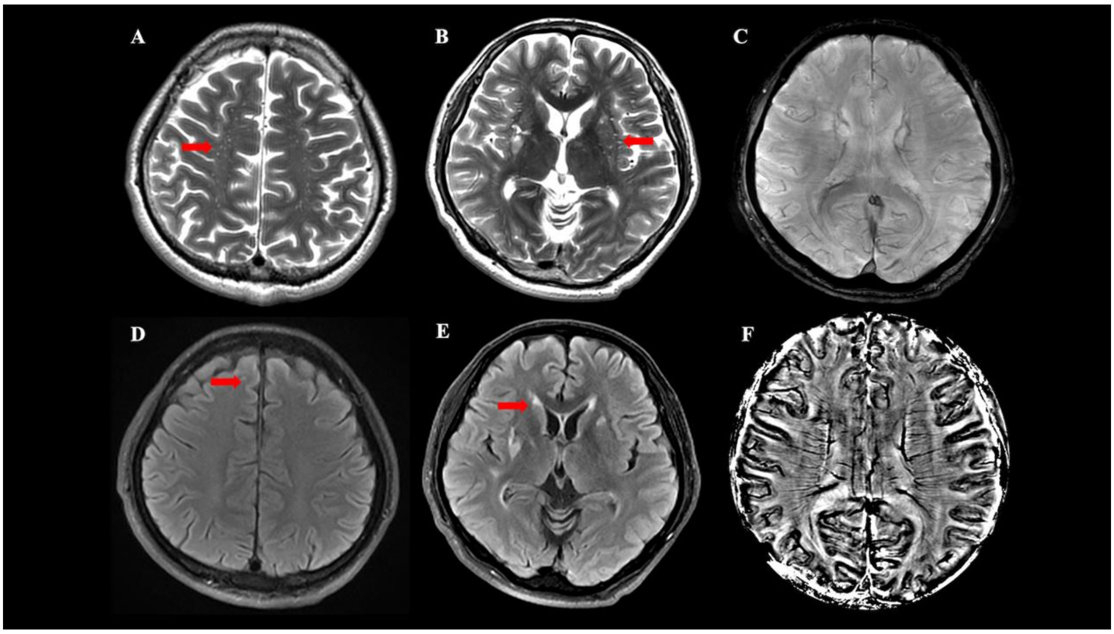

The multimodal magnetic resonance (MR) examination of a 57-year-old man. (A-B) Semi-quantitative score 3 in centrum semiovale white matter and 2 in the basal ganglia (arrow), (C) no hypointensity in SWI sequence, (D) punctate foci in deep white matter in T2- FLAIR sequence, Fazekas score 1 in deep white matter and 1 in periventricular white matter (arrow), (F) in SWI phase image, the DMVs were continuous without interruption, and the signals of parietal region were punctured with hypointensity, the total score was 2. This figure may show that patients with mild SVD burden have a higher probability of having better drainage system for DMV. SWI, susceptibility imaging; FLAIR, fluid attenuated inversion recovery; DMV, deep medullary vein; SVD, small vessel disease.

Supplement Figure 2

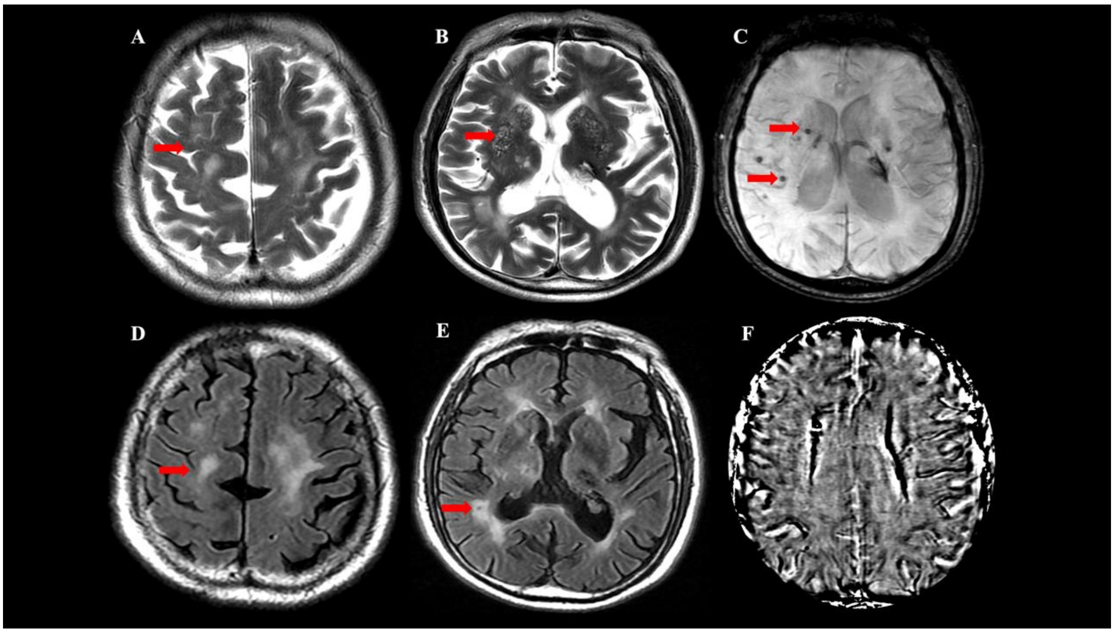

The multimodal magnetic resonance (MR) examination of a 77-year-old woman. (A-B) Semi-quantitative score 2 in centrum semiovale white matter and 4 in the basal ganglia (arrow), (C) multiple punctate hypointensities in SWI sequence, (D) large confluent lesion in deep white matter in T2- FLAIR sequence, Fazekas score 3 and 3 in periventricular white matter (arrow), (F) in SWI phase image, no continuous DMV was seen, the total DMV score was 18. This figure may show that patients with severe SVD burden, especially severe BG-PVS, have a worse deep with matter venous drainage. SWI, susceptibility imaginh; FLAIR, fluid attenuated inversion recovery; DMV, deep medullary vein; SVD, small vessel disease; BG-PVS, basal ganglia perivascular space.
